# Evaluation of Safety and Acceptability of 40 Hz Amplitude-Modulated Auditory Stimulation in Healthy Older People: A Prospective Study from Japan †

**DOI:** 10.3390/healthcare13202638

**Published:** 2025-10-20

**Authors:** Shunsuke Sato, Kazuma Maeda, Hiroki Chinen, Shinzo Hiroi, Keita Tanaka, Eriko Ogura, Hiroki Fukuju, Kentaro Morimoto, Yoshiki Nagatani, Kazuki Takazawa, Taiki Kasai, Yumi Ohta, Manabu Ikeda

**Affiliations:** 1 Health and Counseling Center, Osaka University, Osaka 560-0043, Japan; 2 Department of Psychiatry, Graduate School of Medicine, Osaka University, Osaka 565-0871, Japan; mikeda@psy.med.osaka-u.ac.jp; 3 Shionogi and Co., Ltd., Osaka 541-0045, Japan; kazuma.maeda@shionogi.co.jp (K.M.); hiroki.chinen@shionogi.co.jp (H.C.); shinzo_h@me.com (S.H.); keittana@sbigroup.co.jp (K.T.); eriko.ogura@shionogi.co.jp (E.O.); hiroki.fukuju@shionogi.co.jp (H.F.); kentaro.morimoto@shionogi.co.jp (K.M.); 4 Pixie Dust Technologies, Inc., Tokyo 104-0028, Japan; yoshiki.nagatani@pixiedusttech.com (Y.N.); kazuki.takazawa_p@pixiedusttech.com (K.T.); taiki.kasai@pixiedusttech.com (T.K.); 5 Department of Otorhinolaryngology-Head and Neck Surgery, Graduate School of Medicine, Osaka University, Osaka 565-0871, Japan; yota@ent.med.osaka-u.ac.jp

**Keywords:** 40 Hz, amplitude-modulated sound, auditory stimulation, gamma entrainment, cognition, dementia, healthy older people

## Abstract

**Background/Objectives:** Dysregulated gamma oscillations are associated with cognitive dysfunction. Auditory stimulation at 40 Hz enhances neural activity in brain regions associated with learning, attention, and memory. This study assessed the safety and acceptability of 40 Hz amplitude-modulated auditory stimulation in healthy older people. Auditory stimuli were created using popular songs, where vocals and background music were separated and independently amplitude-modulated at 40 Hz with different modulation depths to generate periodic 40 Hz gamma waveforms. **Methods**: In this open-label, single-arm study, healthy participants aged ≥65 years received 40 Hz amplitude-modulated auditory stimulation daily via a smartphone for 28 days through earphones/headphones. Safety was assessed through adverse event (AE) monitoring and changes in clinical scores for depression, cognitive function, and hearing thresholds. Acceptability was evaluated by adherence rates, listening time, dropout reasons, volume levels, intent for future use, and subjective impressions of the sound source on a 7-point Likert scale. **Results**: Among 28 participants (mean age 69.1 years, 53.6% female), six reported 12 AEs, with six considered device-related (e.g., ear discomfort, jaw pain, musculoskeletal stiffness). The AEs observed were mild or moderate. Scores for cognitive function, depression, and hearing thresholds did not worsen during the study period. Adherence was observed in 96.4%, with 85.7% expressing interest in continuing. Most participants rated the sounds’ unnaturalness between 2 and 3 and discomfort between 1 and 3 on the 7-point Likert scale. **Conclusions**: The intervention was well tolerated and acceptable in study participants, with no major safety concerns identified. Auditory stimulation did not cause severe discomfort or reduce acceptability. Further studies should explore the long-term effects and broader clinical applications.

## 1. Introduction

Currently, there are more than 55 million people living with dementia worldwide, and this number is expected to reach 139 million by 2050 [1]. While disease-modifying drugs for dementia are under development, their therapeutic effects remain limited [2]. Gamma frequency oscillation, characterized by synchronized neuronal activity in the brain (i.e., simultaneous firing of neuronal populations) in the 30–90 Hz range, is associated with higher-order cognitive functions, including attention, memory, and sensory processing [3]. The dysregulation of gamma oscillations is increasingly recognized in the context of aging and cognitive disorders, particularly Alzheimer’s disease (AD) [4]. Noninvasive 40 Hz sensory stimulation offers a promising approach to re-establishing gamma synchrony in the aging brain [5].

Preclinical studies have demonstrated that exposure to gamma frequency sensory stimuli can reduce amyloid-beta plaque levels, modulate microglial activity, and protect against neuronal degeneration [6,7]. Clinical studies investigating 40 Hz sensory stimulation have reported improvements in functional brain connectivity and cognitive function in patients with mild cognitive impairment (MCI) [8] or AD [9,10]. For example, Wang et al. (2024) applied 40 Hz auditory stimulation using music-based sound sources in older adults with MCI [8], while Chan et al. (2022) examined auditory stimulation devices delivering 40 Hz sounds in patients with probable AD [9]. Recently, the OVERTURE study, a randomized double-blind clinical trial, demonstrated the clinical benefits of a 40 Hz audiovisual stimulation device in participants with mild to moderate AD. However, a higher number of patients, although not significant, who used audiovisual stimulation devices experienced treatment-related adverse events (AEs) such as headache and tinnitus compared with those in the sham group [10]. As individuals with cognitive impairment may require sensory stimulation over an extended period, it is important to develop forms of sensory stimulation that are not only acceptable and comfortable but also seamlessly integrated into everyday life [11]. The present study sought to evaluate the safety and acceptability of 40 Hz amplitude-modulated auditory stimulation in healthy older people. A sound source processing method was developed in this study by combining sound source separation and amplitude-modulation technology. We hypothesized that applying uniform modulation to all sound sources within a song, including the lyrics, could make the speech content harder to comprehend and potentially increase discomfort. To address this problem, we used sound source separation to split the original audio into vocals and background music, allowing the independent modulation of each component. We applied 50% modulation to speech and vocals, and 100% to background music (BGM), reducing perceptual load while preserving neural entrainment activity at 40 Hz. A prior study by Nagatani et al. (2023) used electroencephalography (EEG) to investigate the evoked gamma waves in human brains by using a similar amplitude-modulated sound source and demonstrated that 40 Hz-modulated auditory stimulation alone could synchronize participants’ brain waves to 40 Hz [12]. The 40 Hz auditory stimulation can create stronger neural activity in the parietal and prefrontal regions [13], suggesting that the neural response during 40 Hz entrainment may be related to higher-level brain functions such as learning, attention, or memory. Furthermore, the sound source processing method used in this study involved reshaping the envelope of sound signals containing certain pitch or speech information into periodic wave forms, such as sinusoidal waves, at a gamma frequency (40 Hz). Nagatani et al. (2023) [12] reported that square waves strongly induced 40 Hz EEG activity but were associated with higher levels of discomfort, possibly due to the sharp sound breaks. In contrast, sine waves induced weaker 40 Hz EEG activity compared to square waves but were perceived as much more comfortable. The property of sine waves of having simpler oscillation might be more natural to the brain, offering a higher level of perceptual comfort. By integrating sound source separation and amplitude-modulation techniques, we aim to deliver a more comfortable and enduring auditory stimulation method that can complement existing interventions for cognitive health. The findings of this study will contribute valuable insights into the potential of 40 Hz auditory stimulation as a viable tool for enhancing cognitive health in aging populations, potentially paving the way for more accessible and less intrusive treatments for dementia and other cognitive disorders.

## 2. Materials and Methods

### 2.1. Study Design

**Figure 1:**
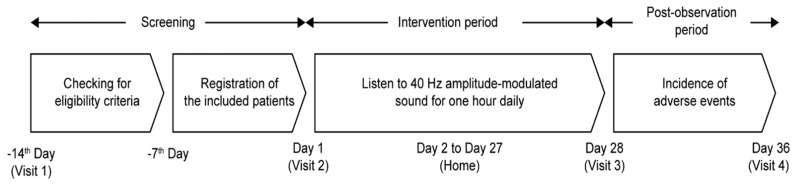
Study design. Screening and eligibility assessments were conducted from Day −14 to Day −7, followed by participant registration on or after Day −7. The 40 Hz amplitude-modulated sound intervention was performed for 28 days starting on Day 1, and adverse events were followed up until Day 36.

This open-label, single-arm, prospective study was conducted from 20 April 2023 to 31 July 2023 (UMIN Study ID: UMIN000050858). All participants underwent a 28-day intervention using 40 Hz amplitude-modulated sound (Figure 1). The 40 Hz amplitude-modulated sound source, generated using a sine-wave modulation, was stored on a smartphone. Participants listened to the sound source using either earphones or headphones provided by the researchers, from which they could choose their preferred device. Initially, at the screening stage, participants were invited to a meeting room where evaluations of their hearing loss, depression, and cognitive function were conducted. Subsequently, the participants were allowed to select a smartphone and earphones/headphones to suit their comfort and needs. The remaining 28-day intervention sessions were performed by participants at their respective homes once a day, and each session lasted for 1 h (approximately 12 Japanese popular songs, each approximately 5 min long, with separate music for each track). The intervention volume was decided by the participants, but the upper limit was restricted to 80 A-weighted sound pressure level (dBA) [14]. From the first day of the intervention, the modulation depth of the auditory stimuli was gradually increased to allow participants to acclimate to the modulated sound environment (Table S1). Vocal tones and BGM, both modulated at 40 Hz, were adjusted separately, and the amplitude modulation of both signals shared the same phase. On Day 1, modulation was set at 0% for vocals and 50% for BGM. BGM modulation increased to 75% on Day 2 and was 100% by Day 3, while vocals were unmodulated. On Day 4, vocal modulation was introduced at 30%. From the fifth day onward, the modulation rate was set at 50% for the vocal track and 100% for BGM. Prespecified endpoints were measured over a period of 30 days. Participants were instructed to record the study endpoints in a structured patient diary. To minimize recall bias, they were encouraged to complete entries on a daily basis. Diaries were submitted weekly to the study investigators. The study protocol was approved by the ethics committee of Osaka University Medical Hospital, Japan. The study was conducted in accordance with the National Legislation and the 1975 Declaration of Helsinki. All participants provided written informed consent before enrollment in the study.

### 2.2. Study Participants

Participants of either gender, aged ≥65 years, who could provide written informed consent were included in this study. Participants with (i) hearing loss, defined as pure-tone audiometry thresholds of >40 dB HL at 2 kHz, >50 dB HL at 4 kHz, or >60 dB HL at 8 kHz, (ii) depression or anxiety disorders (diagnosed with depression or anxiety disorders, or currently taking antidepressant or anxiolytic medications) or those having a 15-item Geriatric Depression Scale (GDS-15) score of ≥5, (iii) dementia (diagnosed with dementia or currently taking dementia-related medications) or those with a Mini-Mental State Examination-Japanese version (MMSE-J) score of ≤27 were excluded from the study.

### 2.3. Study Endpoints

The endpoints of this study included measures of safety and acceptability. Safety endpoints involved the monitoring of AEs occurring during the study period. Key parameters recorded for each AE included its name, severity, causality, onset and resolution dates, outcome, and any treatments administered. Furthermore, changes in the clinical score for depression, cognitive function, and hearing threshold levels were evaluated before and after the intervention period. Depression was evaluated using GDS-15, which has a score range of 0 to 15, whereas cognitive function was measured using MMSE-J, which scores participants between 0 and 30 [15,16]. Hearing thresholds were evaluated before and after the intervention using pure-tone audiometry. For the analysis, the mean hearing threshold was calculated using the three-frequency method, that is, the arithmetic average of thresholds at 500, 1000, and 2000 Hz (3-frequency pure-tone average, dB HL). Acceptability was assessed by examining the number of days the participants were engaged with the sound intervention, i.e., continuation and dropout rates, listening time, reasons for dropout, volume level, subjective impressions of the sound source, and intention of future use. Impressions of the sound source were evaluated using a 7-point Likert scale questionnaire, which captured participants’ perceived unnaturalness (1 = not at all unnatural, 2 = slightly unnatural, 3 = somewhat unnatural, 4 = mild-to-moderate unnatural, 5 = moderately unnatural, 6 = very unnatural, and 7 = extremely unnatural) and discomfort (1 = not at all uncomfortable, 2 = slightly uncomfortable, 3 = somewhat uncomfortable, 4 = mild-to-moderate uncomfortable, 5 = moderately uncomfortable, 6 = very uncomfortable, and 7 = extremely uncomfortable).

### 2.4. Statistical Analysis

The sample size for this study was determined based on two primary considerations: safety and acceptability analysis. For the acceptability analysis, assuming a true adherence rate of 80%, the required sample size was calculated based on a binomial distribution to ensure that the point adherence rate would not fall below 70% with a probability of ≥90% [17]. This yielded a sample size of 20 participants. For the safety analysis, a sample size of 22 participants was determined based on the findings from a related study that examined the safety of a daily 1 h intervention of 40 Hz auditory and visual stimulations over 6 months [9]. A sample size of 22 participants was necessary to capture at least one AE with a frequency of ≥10% with a probability of ≥90%. To accommodate potential dropout during the screening process, a final sample size of 30 participants was determined, which was sufficient to meet both acceptability and safety requirements, assuming a 20% dropout rate. The full analysis set (FAS) consisted of participants who initiated the intervention, regardless of completion status. The incidence and proportion of AEs for which a causal relationship with the intervention could not be ruled out were calculated. The determination was made based on a comprehensive assessment by investigators, considering factors such as temporal relationship, biological plausibility, and the exclusion of alternative causes. AEs were coded using MedDRA version 26.0. Changes in GDS-15, MMSE-J, and hearing levels before and after the intervention were evaluated using the Wilcoxon signed-rank test. The distribution of scores from 1 to 7 for each item in the questionnaire, which evaluated the unnaturalness and discomfort of the sound source, was assessed using the Friedman test. Additionally, the Wilcoxon signed-rank test was used to compare the rank averages between the first and last days. A significance level of *p* < 0.05 was used to determine statistical significance. Statistical analysis was conducted using SPSS Statistics, version 24.0 (IBM SPSS Japan, Tokyo, Japan).

## 3. Results

### 3.1. Patient Disposition

**Figure 2:**
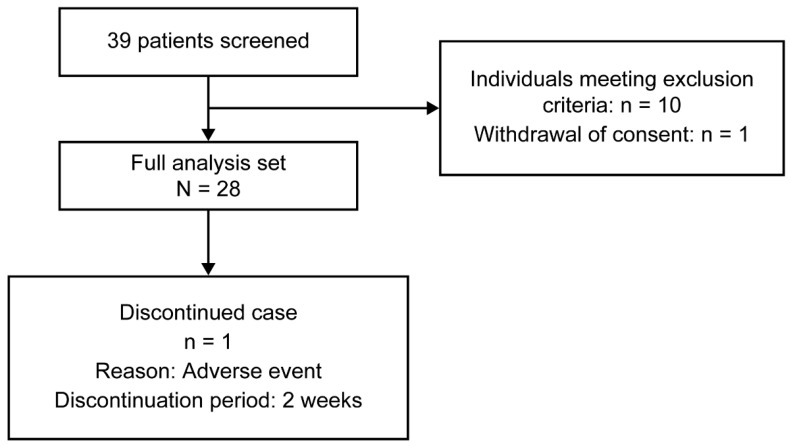
Participant disposition.

Informed consent was obtained from 39 participants. Of these, 11 patients were excluded, 10 due to meeting the exclusion criteria and 1 for withdrawing consent. Although the planned sample size was 30, 28 participants were included in the FAS after exclusions and withdrawal of consent (Figure 2). Among them, 27 completed the study, resulting in a completion rate of 96.4%.

### 3.2. Baseline Characteristics

The mean (standard deviation [SD]) age of the 28 participants in the FAS was 69.1 (4.0) years, with 53.6% being females (Table 1). The mean (SD) listening volume during the entire intervention was 57.8 (3.3) dBA, with values consistently ranging between 57.7 and 58.6 dBA across all days.

### 3.3. Safety

A total of 12 AEs occurred in six participants (21.4%; Table 2). Of these, six events were considered related to the device, including ear discomfort and pain (*n* = 3), musculoskeletal stiffness and jaw pain (*n* = 2), and neck pain (*n* = 1; Table 2). Device-related AEs occurred in two participants (7.1%). One participant experienced multiple device-related AEs, including ear pain, ear discomfort, jaw pain, neck pain, and musculoskeletal stiffness, and ultimately discontinued from the study. Another participant experienced left ear pain and discomfort. The ear pain resolved within 2 days, whereas ear discomfort was unresolved even at the end of the study. However, the participant completed the study protocol without further complications. The AEs were mild to moderate, and no safety evaluation indicators showed deterioration before and after the intervention period.

The mean (SD) depression score was 1.4 (1.1) at baseline and 1.6 (2.1) after the intervention period (Day 28), with no significant difference (*p* = 0.826; Table 3). The mean (SD) cognitive function score indicated an improvement from 29.5 (0.7) at baseline to 29.8 (0.4) after the intervention period (Day 28; *p* = 0.038; Table 3). There were no significant changes in hearing levels during the study period, with the mean (SD) threshold of the right ear changing from 20.2 (6.5) dB HL at baseline to 19.9 (7.1) dB HL after the intervention, and that for the left ear changing from 19.0 (5.9) dB HL to 18.9 (6.4) dB HL (*p* = 0.612 for the right ear, *p* = 0.763 for the left ear; Table 3).

### 3.4. Acceptability

**Figure 3:**
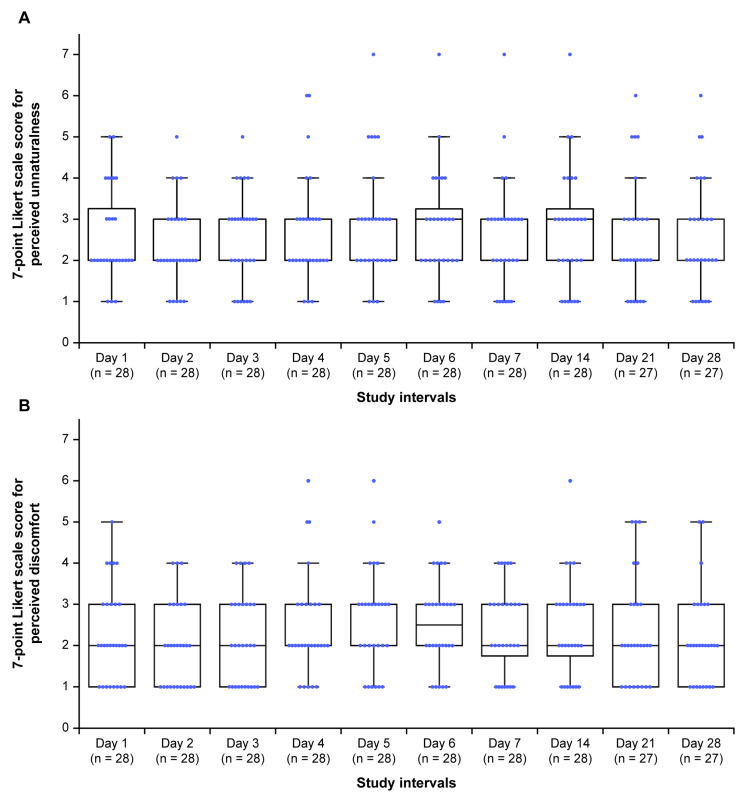
Participants’ perceptions of (**A**) unnaturalness and (**B**) discomfort on a 7-point Likert scale for the 40 Hz modulated sound source during the study period. Horizontal black lines within each box indicate the median, and the boxes represent the interquartile range (25th to 75th percentile). Vertical lines (whiskers) denote adjacent values, i.e., the most extreme values within 1.5 interquartile ranges of the 25th and 75th percentiles. Blue dots represent all individual data points; those lying beyond the whiskers indicate outliers. Within each box, the horizontal black lines denote median values; boxes extend from the 25th to the 75th percentile of each group’s distribution of values; vertical extending lines denote adjacent values (i.e., the most extreme values within 1.5 interquartile range of the 25th and 75th percentile of each group); dots denote observations outside the range of adjacent values. For unnaturalness, 1 = not at all unnatural, 2 = slightly unnatural, 3 = somewhat unnatural, 4 = neutral, 5 = moderately unnatural, 6 = very unnatural, and 7 = extremely unnatural. For discomfort, 1 = not at all uncomfortable, 2 = slightly uncomfortable, 3 = somewhat uncomfortable, 4 = neutral, 5 = moderately uncomfortable, 6 = very uncomfortable, and 7 = extremely uncomfortable.

Listening activities were recorded on a mean (SD) of 98.2% (9.4%) of the days corresponding to the intervention period (Table 4). Participants maintained a listening frequency of 100% from Days 1 to 14, with 27/28 (96.4%) continuing the intervention up to Day 28. Most participants rated the unnaturalness of tones between 2 and 3 on the 7-point Likert scale at different study intervals (Figure 3A). There was no variation in the distribution of scores during the intervention period (*p* = 0.197). Most participants rated the discomfort of tones between 1 and 3 on the 7-point Likert scale from Days 1 to 28 (Figure 3B), with a significant variation in the distribution of scores during the intervention period (*p* = 0.008). Interest in the continuous use of the 40 Hz modulated tones in the future was expressed by 24/28 (85.7%) participants. Of these, 20/24 (83.3%) favored daily use, whereas 16/24 (66.7%) preferred session durations of 30 min to 1 h (Table S2).

## 4. Discussion

This prospective study evaluated the safety and acceptability of 40 Hz amplitude-modulated auditory stimulation in healthy older people in Japan. The auditory intervention demonstrated a manageable safety profile and was well tolerated among the older participants in this study. There were no significant differences in gender distribution among participants, ensuring a balanced representation.

Safety assessments revealed 12 AEs in six participants. Of these, six events were device-related and occurred in two patients. No severe or serious AEs occurred. One participant experienced ear pain, ear discomfort, jaw pain, neck pain, and musculoskeletal stiffness. The pain was localized primarily to areas in contact with the headphones/earphones and may reflect individual sensitivity to prolonged wear or anatomical factors. Due to the persistence of these symptoms, the participant elected to withdraw from the study. In other cases, AEs (both device-related and unrelated) were either resolved during the study period or tolerated well enough to allow continuation. Previous studies have reported withdrawals due to mild AEs such as dizziness and headaches with 40 Hz sensory-stimulation devices [9,18]. In a phase 1 study, Chan et al. (2022) examined gamma frequency sensory stimulation in 10 cognitively normal adults aged 50–100 years [9]. Five participants (50%) reported a total of six AEs: four experienced sleepiness or drowsiness, one reported light sensitivity, and one had a new-onset headache. Cimenser et al. (2021) studied 40 Hz sensory stimulation (1 h daily sessions) in patients with mild to moderate AD over 6 months [19]. Thirteen patients experienced 39 AEs, of which 41% were device-related. Common AEs included headache (26%) and eye irritation (11%). Anxiety, benign positional vertigo, dizziness, ear irritation, fatigue, nasal irritation, and shoulder pain were reported by one patient each. In the current study, the scores for cognitive function, depression, and hearing thresholds did not worsen in the older participants after 28 days of listening to music on the device, which might be another reason underlying the high adherence rate observed in the current study. Although the MMSE-J score increased by 0.3 points (*p* = 0.038), this change is within the measurement error range and is unlikely to have clinical significance. Thus, a 40 Hz auditory-stimulation device may support patient adherence and acceptability, which are important considerations for potential long-term applications. When compared with previous studies (Table 5), auditory 40 Hz stimulation appears to be generally well tolerated, with adverse events typically mild in nature. Studies combining auditory and visual stimulation tended to report a somewhat higher frequency of adverse events. Although serious adverse events have been rare overall, Hajós et al. (2024) reported a case of acute confusion in a multimodal (audiovisual) stimulation study [10]. Importantly, to date, no serious adverse events have been reported in studies using auditory stimulation alone, suggesting that this modality may represent a particularly safe and acceptable approach.

Participants’ adherence to the current study was notably high, with 96.4% completing the intervention period. Participants’ ratings of the unnaturalness and discomfort of the tones, as measured on a 7-point Likert scale, were mostly between 1 and 3 during the 28-day study period. These findings suggest that auditory stimulation was not perceived as very or extremely unnatural or uncomfortable by the participants, thus increasing the acceptability of the intervention. High adherence rates have been previously observed with 40 Hz auditory interventions, with studies indicating that participants engage effectively with these devices when experiencing minimal levels of discomfort [8,12,19]. Interestingly, Nagatani et al. (2023) conducted a systematic comparison of various 40 Hz amplitude-modulated auditory stimuli to investigate their neural and perceptual effects [12]. The results revealed that square-wave modulation induced stronger EEG entrainment but was perceived as more unnatural and uncomfortable, whereas sine wave-modulated sounds were more comfortable despite slightly weaker neural entrainment. The study also reported a positive correlation between the perceived unnaturalness of the sound and discomfort, suggesting that the perceptual qualities of the stimulus play a crucial role in user acceptability. Building on these findings, sine wave amplitude modulation was used in the present study to optimize comfort while maintaining entrainment efficacy. We also employed a sound source separation technique to isolate vocals from background music, which serves as a novelty of the current study. The high adherence rates and low discomfort scores observed in the current study support the validity of this design approach and reinforce the importance of considering perceptual naturalness when developing long-term auditory-stimulation protocols. Wang et al. (2024) reported positive feedback for composite interventions, such as 40 Hz music, which mitigated the discomfort associated with the pure 40 Hz tone [8]. This suggests that auditory stimulation methods that include the 40 Hz component and are perceived as natural may be tolerable to most individuals when administered over a long-term period [12]. These factors warrant further investigation in future studies to optimize the auditory stimulation protocol for maximum comfort. Interestingly, the majority of participants (85.7%) expressed interest in continuing to use the 40 Hz auditory stimulation in the future.

One of the key strengths of this prospective study is that it demonstrates the safety, acceptability, and continuity of the 40 Hz amplitude-modulated sound in healthy older people for a period of approximately 1 month, highlighting the practical applicability of this intervention in real-world settings. Furthermore, it creates an opportunity to explore the potential benefits of 40 Hz auditory stimulation in more targeted populations, such as individuals with dementia or MCI. The sample size, which was carefully determined using statistical power analysis, was deemed sufficient to draw meaningful conclusions. The sample size and patient characteristics of our study align with those of previous studies investigating 40 Hz sensory-stimulation devices [9,19,20,21]. However, our study has some limitations that should be considered when interpreting its findings. First, the single-arm design without a control group and the lack of blinding make it difficult to establish causality or compare the influence of the intervention against other conditions. Future randomized controlled and blinded trials are warranted. Second, the small sample size (*n* = 28) and the stringent exclusion criteria narrow the participant pool, limiting the generalizability of the findings. Nevertheless, when compared with previous studies [8,9,10,19], the safety and acceptability of 40 Hz stimulation have been consistently demonstrated, suggesting that our results may also be generalizable. Third, although six adverse events thought to be related to the device were qualitatively described, their frequency and severity could not be statistically compared due to the limited sample size. Self-reported data, such as sound volume adjustments and impressions of the sound source, may introduce bias or variability, although measures were taken to minimize recall bias. Furthermore, the lack of real-time monitoring during home-based sessions might introduce potential bias and inconsistencies. The maximum age of participants in the current study was 78 years, which may limit the generalizability of our findings to individuals aged 80 years or older. However, previous studies involving older populations, including those over 80 years [8,9,10], also demonstrated good safety and acceptability of 40 Hz sensory stimulation. This suggests that our results may be applicable to even older adults, although further research into this population is warranted. Given that participants experienced slight discomfort and considering that future long-term use is expected in individuals with dementia or MCI to help prevent or delay onset, further efforts are needed to minimize discomfort.

## 5. Conclusions

In this study, the 40 Hz amplitude-modulated sound showed a manageable safety profile and was well tolerated by healthy older people. No new safety concerns were identified. Furthermore, the cognitive function, depression scores, and hearing thresholds did not worsen even after 28 days of listening to the device. These findings suggest that the 40 Hz amplitude-modulated sound could be a viable noninvasive therapeutic option. Future studies should explore its efficacy in improving cognitive function, particularly in patients with dementia or MCI.

## Figures and Tables

**Table 1:** Baseline characteristics.

Characteristics	Full Analysis Set (*n* = 28)
Age, years	Mean (SD): 69.1 (4)
	Median (min, max): 67.5 (65, 78)
Gender, *n* (%)	Male: 13 (46.4), Female: 15 (53.6)
Listening volume, dBA	Mean (SD): 57.8 (3.3)
	Median (min, max): 57.4 (53.7, 66.6)

dBA, A-weighted sound pressure level; max, maximum; min, minimum; SD, standard deviation.

**Table 2:** Frequency of individual AEs (*n* = 28).

SOC	PT	Any AE, *n* (%)	Causal Relationship, *n* (%)
Nervous system disorders	Headache	2 (7.1)	0 (0)
	Increased depression scale score	2 (7.1)	0 (0)
Ear and labyrinth disorders	Ear pain	2 (7.1)	2 (7.1)
	Ear discomfort	2 (7.1)	1 (3.6)
	Low-frequency hearing loss	1 (3.6)	0 (0)
Musculoskeletal and connective tissue disorders	Jaw pain	1 (3.6)	1 (3.6)
	Musculoskeletal stiffness	1 (3.6)	1 (3.6)
	Neck pain	1 (3.6)	1 (3.6)
Total	-	6 (21.4)	2 (7.1)

AE, adverse event; PT, preferred term; SOC, system organ class.

**Table 3:** Changes in cognition, depression, and hearing threshold scores.

	Baseline (*n* = 28)	Day 28 (*n* = 27)	Mean Difference (*n* = 27)	*p*-Value *
Mean (SD) MMSE-J score	29.5 (0.7)	29.8 (0.4)	0.4 (0.9)	0.038
Mean (SD) GDS-15 score	1.4 (1.1)	1.6 (2.1)	0.1 (1.7)	0.826
Mean (SD) hearing threshold for the right ear, dB HL	20.2 (6.5)	19.9 (7.1)	−0.4 (2.2)	0.612
Mean (SD) hearing threshold for the left ear, dB HL	19.0 (5.9)	18.9 (6.4)	−0.1 (1.9)	0.763

Hearing threshold, the average of thresholds at 500, 1000, and 2000 Hz in pure tone audiometry; dB HL, hearing level; GDS-15, 15-item Geriatric Depression Scale; MMSE-J, Mini-Mental State Examination-Japanese version; SD, standard deviation. * *p*-value was determined using Wilcoxon signed-rank test. Baseline values are presented for all 28 participants, including one participant who discontinued the study after Day 14. Day 28 values and mean differences are based on the 27 participants who completed the intervention.

**Table 4:** Listening history of participants.

	Full Analysis Set (*n* = 28)
Frequency of listening to sound source, **%**	
Mean (SD)	98.2 (9.4)
Median (min, max)	100 (50, 100)
Participants engaged in listening to the sound source, *n* (%)	
Day 1	28 (100)
Day 2	28 (100)
Day 3	28 (100)
Day 4	28 (100)
Day 5	28 (100)
Day 6	28 (100)
Day 7	28 (100)
Day 14	28 (100)
Day 21	27 (96.4)
Day 28	27 (96.4)

Max, maximum; min, minimum; SD, standard deviation. Frequency of listening to sound source: (each participant) = (number of days with listening)/(number of days corresponding to the intervention period) × 100.

**Table 5:** Comparison of the present study with previous clinical studies on 40 Hz stimulation.

(**A**) Auditory-only stimulation studies							
**Study (Year)**	**Population/Age Range (Mean ± SD)**	***n***	**Stimulus & Modality**	**Dose/Duration**	**Device/Delivery**	**Adverse Events/Safety**	**Acceptability/Compliance**
Wang et al. (2024) [8]	Older adults with MCI, 55–81 yrs (68.0 ± 7.1)	25	40 Hz auditory stimulation (music-based)	1 h/day × 4 weeks per condition (crossover)	Tablet or MP3/Headphones at home	40 Hz sound: discomfort, irritation, residual tinnitus (up to 1 h), headache, protocol deviations (sleeping/TV); 40 Hz music: more tolerable	Music: highly favorable; 40 Hz sound: mixed/low; 40 Hz music: improved acceptability; quantitative adherence not yet reported
Present study (this work)	Healthy older adults, 65–78 (mean 69.1 ± 4.0)	28	40 Hz amplitude-modulated auditory (music, vocals/BGM separated)	1 h/day × 28 days	Smartphone + headphones or earphones	12 AEs in 6 participants; 6 device-related (ear pain, discomfort, jaw/neck issues)	96.4% completion; 85.7% interest in future
(**B**) Audiovisual stimulation studies							
**Study (Year)**	**Population/Age Range (Mean ± SD)**	***n***	**Stimulus & Modality**	**Dose/Duration**	**Device/Delivery**	**Adverse Events/Safety**	**Acceptability/Compliance**
Cimenser et al. (2021) [19]	Mild–moderate AD, ≥55 (Active: 66.5 ± 8.0; Control: 73.5 ± 6.6)	22 (Active: 14; Control: 8)	40 Hz auditory + visual sensory stimulation (closed-eyes condition)	1 h/day × 6 months	Gamma sensory stimulation device (Cognito Therapeutics)/Eye-set + Headphones	Device-related AEs in 42% (mostly mild; 2.5% moderate, 2.5% severe); 1 discontinuation per group	High adherence: 91% average over 6 months
Chan et al. (2022) [9]	Mild probable AD patients, 60–85 yrs (Active: 77.6 ± 7.5; Control: 71.2 ± 8.2)	15 (Active: 8; Control: 7)	40 Hz audiovisual stimulation (flickering LED light + 40 Hz auditory clicks) vs. control (constant white light + white noise)	1 h/day × 3–4 months	Home-based LED light panel + speaker/delivered simultaneously to eyes and ears	Well tolerated; no serious adverse events; no epileptiform discharges on EEG	High compliance: mean usage −87% (active) vs. −91% (control), no group difference
Hajós et al. (2024) [10]	Mild to moderate AD, age ≥ 50 (Active: 69.7 ± 8.0; Control: 75.6 ± 10.0)	70 (Active: 43; Sham: 27)	40 Hz audiovisual stimulation (goggles + headphones) vs. sham	1 h/day × 6 months	Home-based CogTx-001 device/Visual headset + headphones	TEAEs: 65% active vs. 71% sham; TRAEs: 35% active vs. 25% sham; common: headache (22%), tinnitus (15%); 1 severe AE (acute confusion)	High adherence: 81% active vs. 92% sham

SD, standard deviation; MCI, mild cognitive impairment; AD, Alzheimer’s disease; TEAEs, Treatment-Emergent Adverse Events; TRAES, Treatment-Related Adverse Events.

## Data Availability

The datasets generated during and/or analyzed during the current study are available from the corresponding author on reasonable request. The data are not publicly available due to [restrictions imposed by the ethics committee that approved the study].

## Supplementary Materials

The following supporting information can be downloaded at: https://www.mdpi.com/article/10.3390/healthcare13202638/s1, Supplementary File: Table S1. Progression of modulation depth and hearing duration during the study period; Table S2. Participants’ opinion of the sound source after study completion.
