# Supplementary material for: Evaluation of Safety and Acceptability of 40 Hz Amplitude-Modulated Auditory Stimulation in Healthy Older People: A Prospective Study from Japan
Source: Healthcare (Basel). 2025 Oct 20;13(20):2638. doi: 10.3390/healthcare13202638 (PMC12564345; doi:10.3390/healthcare13202638)
Supplement: Supplementary file 1 [file healthcare-13-02638-s001.zip › healthcare-3880769-supplementary.pdf]

SUPPLEMENTARY FILE

Title

Evaluation of safety and acceptability of 40 Hz amplitude-modulated auditory stimulation in healthy older people: A prospective study from Japan

Authors and affiliations

Shunsuke Sato[1,2*], Kazuma Maeda[3], Hiroki Chinen[3], Shinzou Hiroi[3], Keita Tanaka[3], Eriko Ogura[3], Hiroki Fukuju[3], Kentaro Morimoto[3], Yoshiki Nagatani[4], Kazuki Takazawa[4], Taiki Kasai[4], Yumi Ohta[5], Manabu Ikeda[2]

[1]Health and Counseling Center, Osaka University, Osaka 560-0043, Japan

[2]Department of Psychiatry, Graduate School of Medicine, Osaka University, Osaka 565-0871, Japan

[3]Shionogi and Co., Ltd., Osaka 541-0045, Japan

[4]Pixie Dust Technologies, Inc., Tokyo 104-0028, Japan

[5]Department of Otorhinolaryngology-Head and Neck Surgery, Graduate School of Medicine, Osaka University, Osaka 565-0871, Japan

*Corresponding author

Shunsuke Sato, M.D., Ph.D.

Health and Counselling Centre

Osaka University

1-17 Machikaneyama-cho, Toyonaka, Osaka 560-0043, Japan

Department of Psychiatry, Graduate School of Medicine

Osaka University

2 Chome-2 Yamadaoka, Suita, Osaka 565-0871, Japan

Telephone: +81-6-6850-6038

Email address: s.sato1815@gmail.com

ORCID ID: 0000-0002-1378-4428

Table S1. Progression of modulation depth and hearing duration during study period

Day (relative to the starting day of the intervention)	Visit type	Vocal modulation (%)	BGM modulation (%)	Hearing duration
-14	Visit 1	50	100	15 minutes
1	Visit 2	0	50	1 hour
2	Home	0	75	1 hour
3	Home	0	100	1 hour
4	Home	30	100	1 hour
5–28	Home	50	100	1 hour*

*The sound source was heard for 1 hour daily from Day 5 to Day 28
BGM, background music

Table S2. Participant's opinion of the sound source after study completion

	Day 28 (N = 28)
Question 1: Do you want to continue listening to 40 Hz-modulated tones after tomorrow?	
Yes	24 (85.7)
No	4 (14.3)
Question 2: If you choose yes (for question 1), please select the acceptable time and frequency of listening from the following options:	
Time of listening	
30 minutes	3 (12.5)
30 minutes to 1 hour	16 (66.7)
1 hour to 2 hours	5 (20.8)
2 hours or more	0 (0)
Frequency of listening	
Daily	20 (83.3)
Approximately once every few days	4 (16.7)
Once a week	0 (0)
Approximately once a month	0 (0)
Question 3: If in the future, 40 Hz modulated tones are proven to prevent dementia, do you think continuous listening is required for effectiveness?	
Yes	27 (96.4)
No	1 (3.6)
Question 4: If you choose yes (for question 3), please select the acceptable time and frequency of listening from the following options:	

Time of listening	
30 minutes	4 (14.8)
30 minutes to 1 hour	18 (66.7)
1 hour to 2 hours	5 (18.5)
2 hours or more	0 (0)
Frequency of listening	
Daily	24 (88.9)
Approximately once every few days	3 (11.1)
Once a week	0 (0)
Approximately once a month	0 (0)

The data values in this table are represented as n (%)
